# Biomarkers Assessing Endothelial Dysfunction in Alzheimer’s Disease

**DOI:** 10.3390/cells12060962

**Published:** 2023-03-22

**Authors:** Antía Custodia, Marta Aramburu-Núñez, Mariña Rodríguez-Arrizabalaga, Juan Manuel Pías-Peleteiro, Laura Vázquez-Vázquez, Javier Camino-Castiñeiras, José Manuel Aldrey, José Castillo, Alberto Ouro, Tomás Sobrino, Daniel Romaus-Sanjurjo

**Affiliations:** 1 NeuroAging Group (NEURAL), Clinical Neurosciences Research Laboratory (LINC), Health Research Institute of Santiago de Compostela (IDIS), 15706 Santiago de Compostela, Spain; 2 Centro de Investigación Biomédica en Red en Enfermedades Neurodegenerativas (CIBERNED), Instituto de Salud Carlos III, 28029 Madrid, Spain; 3 Neuroimaging and Biotechnology Laboratory (NOBEL), Clinical Neurosciences Research Laboratory (LINC), Health Research Institute of Santiago de Compostela (IDIS), 15706 Santiago de Compostela, Spain

**Keywords:** albumin, Alzheimer’s disease, cell adhesion molecules, endothelial dysfunction, endothelin-1, EPCs, metalloproteinases, neuroimaging, vascular alteration, VEGF

## Abstract

Alzheimer’s disease (AD) is the most common degenerative disorder in the elderly in developed countries. Currently, growing evidence is pointing at endothelial dysfunction as a key player in the cognitive decline course of AD. As a main component of the blood–brain barrier (BBB), the dysfunction of endothelial cells driven by vascular risk factors associated with AD allows the passage of toxic substances to the cerebral parenchyma, producing chronic hypoperfusion that eventually causes an inflammatory and neurotoxic response. In this process, the levels of several biomarkers are disrupted, such as an increase in adhesion molecules that allow the passage of leukocytes to the cerebral parenchyma, increasing the permeability of the BBB; moreover, other vascular players, including endothelin-1, also mediate artery inflammation. As a consequence of the disruption of the BBB, a progressive neuroinflammatory response is produced that, added to the astrogliosis, eventually triggers neuronal degeneration (possibly responsible for cognitive deterioration). Recently, new molecules have been proposed as early biomarkers for endothelial dysfunction that can constitute new therapeutic targets as well as early diagnostic and prognostic markers for AD.

## 1. Introduction

Alzheimer’s disease (AD) is the main neurodegenerative disease leading to dementia and cognitive impairment in the elderly worldwide. The classic pathophysiological hallmarks of AD are extracellular β-amyloid (Aβ) plaques and intracellular tau tangles, which eventually lead to the impairment of cognitive functions. These features progress slowly and are asymptomatic in the first stages of the disease. In fact, this fact hinders an acute premortem diagnosis if not aided by biological markers, as AD symptomatology may share similarities with other causes of dementia.

Besides cellular alterations, AD is also characterized by the presence of several vascular alterations, including small infarcts, or lacunes, due to the occlusion of branches of cerebral arteries, increases in the number of atrophic vessels and amount of vascular tortuosity (abnormal twists and turns in vessels), and decreases in microvascular density and length. Indeed, such brain vascular-associated alterations underlie many pathophysiological mechanisms of AD [1,2]. Accordingly, the two-hit vascular hypothesis points at the initial damage in cerebral vasculature (hit one) as the inducer of the accumulation of β-amyloid in the brain (hit two) [3]. Remarkably, cerebral blood–brain barrier (BBB) leakage and microbleeds are associated with cognitive decline in patients with mild cognitive impairment (MCI) and early AD, which opens the door to search for new biomarkers allowing for the diagnosis of AD before symptoms start. Importantly, many recent studies assessed the relationship between AD and stroke, another vascular-related neurological disease with a worldwide impact [4,5]. In this regard, a meta-analysis revealed that all stroke subtypes significantly increase the risk of developing AD [4]; and, more recently, that several differentially expressed genes and cellular pathways are shared by both stroke and AD [5]. Furthermore, our group has highlighted that higher numbers of circulating endothelial progenitor cells (EPCs) within the first week following stroke have a positive impact on functional outcome [6,7,8,9], and several studies point to EPCs as a beneficial target for AD [1]. Altogether, EPCs may offer a new target to find a treatment for AD based on their recent molecular and genetic connections.

In this review, we will focus on vascular-related biomarkers with the potential to become indicators of either the beginning and/or progression of AD; therefore, we will review the latest evidence regarding the relationship between vascular damage and AD, as well as how this interconnection has an impact on AD pathophysiology.

## 2. Vascular Alterations in AD Brains

The presence of vascular alterations entails an increased risk of developing dementia [10]. A meta-analysis of 2856 patients showed that a 60% prevalence of dementia was present in patients with macroinfarcts and lacunar disease; this percentage diminishes to 56% in small-vessel disease patients, and ranges from 57 to 70% in the presence of microinfarcts (depending on the number of lesions) [10]. More precisely, a post-mortem study found that 80% of AD patients had vascular pathology (e.g., presence of large infarcts, lacunes and multiple microinfarcts, hemorrhages, atherosclerosis, and arteriosclerosis), which has a higher prevalence than in other neurodegenerative diseases [11]. Furthermore, the presence of cerebrovascular disease, any condition affecting blood flow and blood vessel structure negatively, increases the risk of dementia in AD, with a more prominent effect in the earlier stages of the pathology [11]. Although microvascular alterations occur during normal ageing, they are especially prominent in neurodegenerative diseases, such as AD [12]. Some of these alterations include a decrease in the microvascular density, and the increase in both the number of atrophic vessels and string capillaries in AD patients [12,13,14]. In addition, fusiform dilatations, tortuosity, abnormal branching and fusions, as well as a reduction of the total length were detected in capillaries from AD samples [13,15,16].

**Figure 1:**
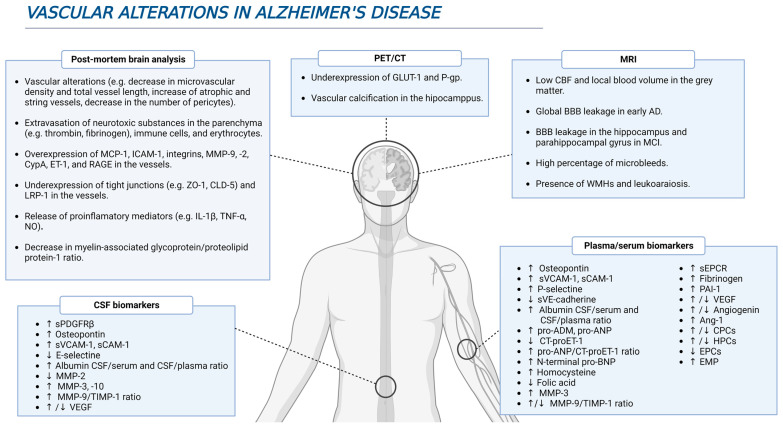
Several vascular-related alterations during AD. Different studies using in vivo and postmortem approaches have brought to light multiple evidence highlighting a feasible role of vascular dysfunction in AD pathophysiology.

The analysis of brain samples from AD patients has yielded a large amount of evidence indicating the presence of vascular alterations (Figure 1). For example, immunohistochemical analysis has detected the presence of potentially neurotoxic substances in the parenchyma due to their extravasation through the BBB, such as prothrombin, thrombin, fibrinogen, fibrin, albumin, and immunoglobulins [15,17,18,19,20,21]. In the specific case of prothrombin, levels of this protein in the prefrontal cortex were higher as the Braak stage increased, pointing to a greater damage of the BBB throughout the disease progression [20]. Furthermore, there is an extravasation and accumulation of immune cells in AD brains that are mediated by the overexpression of molecules such as monocyte chemoattractant protein-1 (MCP-1), intercellular adhesion molecule 1 (ICAM-1), and integrins, promoting neuroinflammation as well as BBB damage [17,22,23,24,25,26]. In addition, the release of many pro-inflammatory mediators, such as interleukin (IL)-1β (IL-1β), IL-6, IL-8, tumor necrosis factor α (TNF-α), TWEAK, and transforming growth factor β (TGF-β), into the vessels strengthen such marked neuroinflammation [17,22,27,28]. Finally, accumulation of erythrocytes and hemosiderin deposits was also detected in AD brain samples, and this is consistent with the presence of microbleeds detected by magnetic resonance imaging (MRI) [29].

Pericytes are one of the most important cells in the brain as their role is keeping the structure of the BBB intact. In AD patients, there is a ~60% decrease in the number of pericytes in the capillaries of the cortex, and a 33% one in the hippocampus compared to controls [19]. Accordingly, decreased pericyte marker platelet-derived growth factor receptor-β (PDGFRβ) and marked degeneration of pericytes were observed [13,15,19]. Both signals would explain the increase in BBB leakage, since the presence of soluble PDGFRβ (sPDGFRβ) in cerebrospinal fluid (CSF) is a marker of BBB leakage [30]. Tight junctions (TJ) are fundamental elements for the correct maintenance of the BBB that connect endothelial cells with each other [31]. Three important proteins of TJ are claudin-5, occludin, and *zonula occludens* 1 (ZO-1), all of which are decreased in cortical areas of AD patients [32,33,34].

Reduced levels of claudin-5 and occludin were correlated with higher Braak states, Aβ_40_ levels, and loss of synaptic markers, such as synaptophysin [34]. Interestingly, Aβ deposits downregulated the expression of occludin and ZO-1 [32]. Furthermore, the decrease in TJ levels could be also explained by the overexpression of cyclophilin A (CypA), metalloprotease-9 (MMP-9), and MMP-2, since this pathway promotes the TJ degradation [15,35]. Furthermore, increased levels of endothelin-1 (ET-1), a potent vasoconstrictor, are also associated with BBB leakage [36].

Alteration and damage of the BBB not only induce the extravasation of substances but also tissue hypoperfusion. Consistent with data from neuroimaging studies, biochemical evidence of cerebral hypoperfusion was found in AD brain tissue. For example, a decreased myelin-associated glycoprotein/proteolipid protein-1 ratio, which indicates hypoperfusion, was detected in AD brains [21,36]. Moreover, this decrease was associated positively with PDGFRβ levels, but negatively with ET-1 levels, Braak stages, Aβ, and plaque levels [21,36]. Furthermore, several molecular changes have also been observed in the cerebral endothelium of AD patients, many of which eventually lead to an increase in brain Aβ accumulation. For example, there is a lower expression of the lipoprotein receptor-related protein 1 (LRP-1) and a higher expression of the receptor for advanced glycation end products (RAGE) in the brain endothelium [32,37,38]. LRP-1 is a transporter that together with P-glycoprotein (P-gp) is involved in Aβ clearance, while RAGE is a receptor that uptakes Aβ from the circulation into the parenchyma [32,37,38].

Overall, there are robust data from AD brains pointing at a clear relationship between the vascular system and AD; however, most of these results come from post-mortem analysis, impeding their use for AD diagnosis but not for discovering new therapeutic targets; thus, it is necessary to study these molecules in the early stages of the disease and assess their potential to be used as biomarkers of AD beginning and/or evolution. In the following sections, we will review and discuss the most recent and relevant works addressing the role of these molecules as potential AD biomarkers.

## 3. Neuroimaging to Find Vascular Biomarkers in AD

Several neuroimaging techniques are being used to detect vascular alterations in AD, such as positron emission tomography (PET), computed tomography (CT), and/or MRI. PET can detect changes in the activity of many transporters and receptors present in the BBB that are essential for the maintenance of its homeostasis. In this sense, PET can detect reduced expressions of the glucose transporter 1 (GLUT-1) and P-gp in the BBB of MCI and AD patients by using different tracers, such as (18)F-FDG and [11C]-verapamil, which reveal alterations in both energy metabolism and Aβ clearance [39,40,41]. Regarding CT, it allowed the detection of vascular calcifications in the hippocampus of AD patients, which have been associated with increased cognitive impairment [42]. Moreover, it has been suggested that vascular microcalcifications may be associated with the presence of hyperphosphorylated tau (p-tau) in neurons [43].

On the other hand, MRI is a key neuroimaging approach for the detection of cerebrovascular alterations in AD, such as BBB leakage, microbleedings, and white-matter hyperintensities (WMH). Moreover, the arterial spin labeling MRI (ASL-MRI) allows the measurement of the cerebral blood flow (CBF). In early AD, lower CBF and local blood volume were detected in the grey matter, being the low CBF correlated with increased BBB leakage [44]. In this line, dynamic contrast-enhanced MRI (DCE-MRI) is the main technique to measure BBB leakage by using gadolinium-based contrast agents. Similar to CBF, a global leakage of the BBB was detected in early AD, with a greater leakage in the deep grey matter and cortex that was significantly associated with a lower score on the Mini-Mental State Examination (MMSE) cognitive test [45]. Interestingly, MCI patients showed BBB leakage in the hippocampus (subfield CA1, CA3, and dentate gyrus) and parahippocampal gyrus independently of both tau and Aβ concentrations in CSF [46]. Indeed, higher leakage in these regions significantly predicts cognitive impairment [46]. Recently, a study assessed the use of new MRI-based technology to study the BBB permeability in MCI patients without contrast agents, by analyzing the leakage of small molecules, such as water [47]. Lin and colleagues showed that AD patients had increased BBB permeability compared to controls, but only for small molecules and not large ones such as albumin [47]. Remarkably, this BBB leakage was correlated with AD markers in CSF (Aβ and p-tau) and worse episodic memory. Overall, it seems that the BBB leakage already starts in important memory-related regions during MCI, and this evolves into global BBB leakage in early AD. It is also important to mention that BBB leakage in the hippocampus also occurs in aging in an age-dependent manner [48].

Finally, MRI technology is particularly useful to study the presence of microbleeds through the acquisition of T2*-weighted sequences that detect iron deposits and, consequently, hemosiderin deposits [49]. Both AD and MCI patients present a higher percentage of microbleeds compared to age-matched and cognitively healthy controls [49]. In the case of MCI, the prevalence of these lesions ranges between 24.3–41%, while the prevalence varies between 40–48% in AD subjects [50,51]. These microbleeds mostly occur in lobar regions, although they have also been detected in non-lobar regions, such as basal ganglia or thalamus [49,50,51]. Another damage associated with cerebrovascular dysfunction is the presence of white-matter lesions detected as hyperintensities (WMHs), which eventually evolved to leukoaraiosis. Normally asymptomatic, leukoaraiosis precedes neurological diseases, such as AD and stroke [52,53]. Of interest, a soluble form of TWEAK (sTWEAK), a well-known pro-inflammatory biomarker, has been recently described as a leukoaraiosis biomarker in stroke [53], although this has not yet been addressed in AD. WMHs in the fornix, splenium of the corpus callosum, temporal lobe, and subcortical multilobar white matter, are associated with greater cognitive impairment [54,55]. Interestingly, WMHs volume is more strongly associated with preclinical AD than other biomarkers of neurodegeneration, and it also shows a correlation with brain deposits of Aβ [56]. In AD patients, there is a greater presence of these lesions than in age-matched controls [54,55,57].

## 4. Vascular Biomarkers in AD

In this section, we will review the latest evidence and relevant works assessing the potential of several vascular-related molecules to become AD biomarkers.

### 4.1. Vascular Inflammation

#### 4.1.1. Osteopontin (OPN)

**Figure 2:**
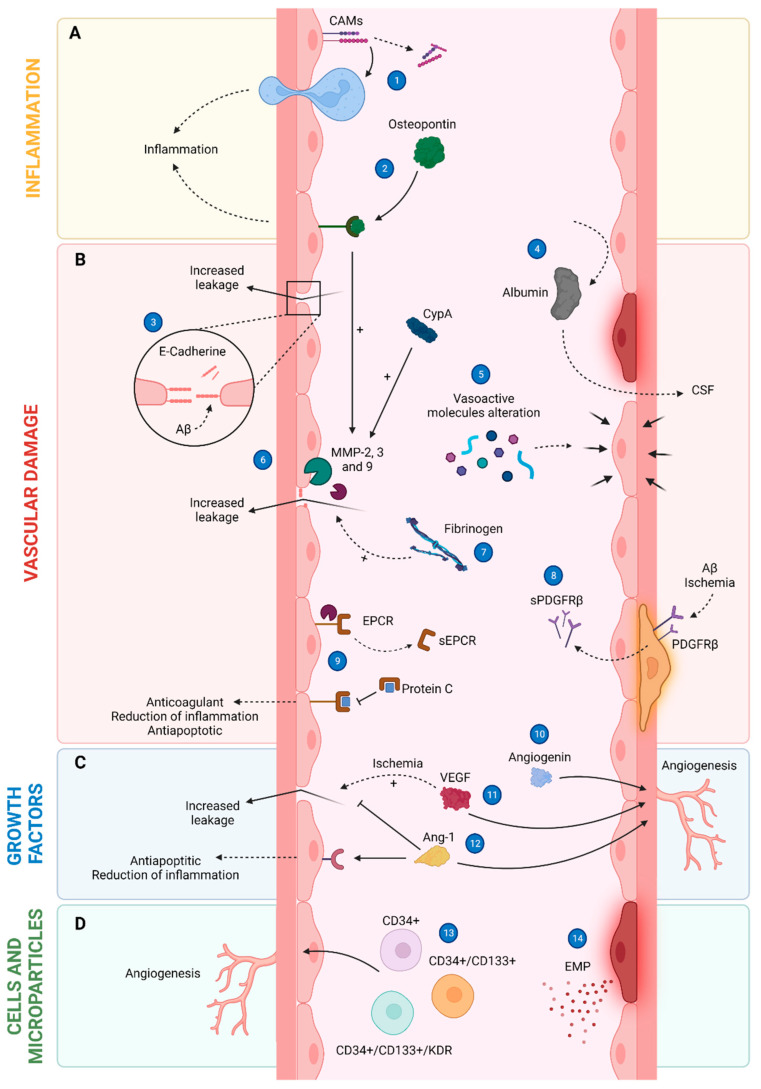
Several cellular pathways underlying endothelial dysfunction. (**A**) Inflammation: CAMs are released from the endothelial membrane in their soluble form, and this process is involved in immune cell extravasation causing vascular dysfunction and inflammation (1). OPN acting through its receptor causes brain inflammation (2). (**B**) Vascular damage: Aβ induces the release of the extracellular domain of VE-cadherin, increasing leakage due to vascular damage (3). The presence of albumin in both the CSF and the brain means BBB damage as it is a protein only synthesized in the liver, thus, implying diffusion from the blood to the brain tissue (4). The action of several vasoactive molecules may cause either vasoconstriction or vasodilatation during AD (5). Several MMPs provoke BBB leakage by inducing the cleavage of tight junction proteins and basal lamina components (6), as well as high levels of fibrinogen (7). Under ischemic conditions or the presence of the Aβ peptide, there is a release of the soluble fragment of the PDGFRβ from pericytes, indicating damage (8). The soluble EPCR competes with the cellular EPCR for protein C binding, having an indirect pro-apoptotic, pro-coagulant, and pro-inflammatory function (9). (**C**) Growth factors: angiogenin (10), VEGF (11), and Ang-1 (12) promote angiogenesis; besides, VEGF can promote BBB leakage in the brain under ischemic conditions, an event that can be diminished by Ang-1. (**D**) Cells and microparticles: CPCs (CD34^+^) are a group of cells involved in tissue maintenance and repair, including HPCs (CD34^+^/CD133^+^) and EPCs (CD34^+^/CD133^+^/KDR), that are associated with vascular repair (13). Elevated amounts of endothelial-derived EMPs usually indicate endothelial injury (14).

OPN is an inflammatory biomarker whose expression is increased under conditions of stress, tissue damage, cerebral ischemia, and vascular diseases [58,59,60,61] (Figure 2). Importantly, higher levels of OPN are closely related to chronic inflammatory diseases such as vascular inflammation and/or atherosclerosis [61]. It is generally accepted that OPN plays a role in inflammation-associated neurological diseases, including AD. Interestingly, MCI patients who eventually developed AD showed elevated levels of OPN in CSF samples [59,60], which aligns with data from AD patients, either in the absence or presence of cardiovascular disease [58,59]. Curiously, AD patients with a recent diagnosis had higher levels of OPN in plasma and CSF samples than those diagnosed more than two years before [59,60]. Importantly, the diagnostic sensitivity of OPN to detect AD was higher in the absence of cerebrovascular disease than in its presence, 92.3% vs. 81% respectively [58]. In addition, OPN levels were positively associated with neuroimaging markers of brain atrophy and worse global cognition, verbal memory, visual memory, executive function, attention, language, visual-motor speed, and visuoconstruction [58]; however, other studies found that elevated levels of OPN in the CSF of AD patients were significantly associated with less cognitive impairment [59,60] (Table 1); therefore, OPN levels in plasma and CSF may have a different correlation with cognitive status depending on the stage of the disease; thus, further studies are mandatory to evaluate whether OPN levels could be used as complementary biomarkers of AD progression.

#### 4.1.2. Cell Adhesion Molecules (CAMs)

CAMs are glycoproteins localized on the cell surface that mediate adhesion between cells and/or between cells and the extracellular matrix. One of their functions is to mediate the adhesion and trafficking of leukocytes through the vasculature for their subsequent extravasation into damaged or inflamed tissue. There are four types of CAMs: cadherins, integrins, selectins, and the immunoglobulin superfamily. Interestingly, CAMs have been linked to vascular disease and, in fact, they are released from the endothelial membrane in their soluble form and can be detected in blood as biomarkers [62,63] (Figure 2). Vascular cell adhesion molecule-1 (VCAM-1) and intracellular cell adhesion molecule-1 (ICAM-1), involved in the process of immune cell extravasation, are considered biomarkers of vascular dysfunction and inflammation [60,64]. Significantly elevated levels of VCAM-1 and ICAM-1 have been detected in both plasma and CSF samples from AD subjects compared to controls and MCI patients [17,22,23,60,64,65,66]. Interestingly, both protein concentrations were associated with elevated CSF levels of total tau (T-tau) and p-tau, and Aβ pathology [66], as well as with BBB permeability (measured by CSF/serum albumin ratio) [65]. Remarkably, VCAM-1 and ICAM-1 were strongly associated with a more rapid progression of cognitive impairment in AD due to their association with a longitudinal increase in CDR-SB scores [62,66]. Overall, this could indicate alterations in the vasculature since increased plasma levels of these molecules in other pathological settings, such as peripheral arterial disease, are positively correlated with microvascular impaired endothelium-dependent vasodilation [67]; however, there were also controversial results showing lower levels of VCAM-1 in CSF samples from AD patients compared to controls [65]. Besides VCAM-1 and ICAM-1, AD has also been linked to other CAMs, such as selectins [68,69]. In this way, levels of E-selectin in CSF samples are inversely correlated with the ratio of total-tau/Aβ_42_ in CSF [68], and elevated P-selectin levels were detected in the blood of AD patients compared to controls [69] (Table 1). Therefore, most of the evidence appears to support the potential use of these molecules as markers for the progression of AD, although more studies are needed to fully confirm such a role as there is some controversy surrounding it.

**Table 1:** Main studies assessing biomarkers for vascular inflammation in AD.

Biomarker	Publications	Results
OPN	- Chai et al. [57]	- AD patients show elevated levels of OPN, and those were positively associated with brain atrophy, worse global cognition, verbal memory, visual memory, executive function, attention, language, visual-motor speed, and visuoconstruction.
	- Comi et al. [58], and Sun et al. [59]	- MCI patients developing AD and recently diagnosed AD patients had higher levels of OPN, and this was significantly associated with less cognitive impairment.
CAMs	- Janelidze et al. [66]	- A positive association between VCAM-1 and ICAM-1 levels and CSF levels of total tau (T-tau) and p-tau, and Aβ pathology. A higher increase in CDR-SB scores.
	- Li et al. [68]	- Inverse correlation between levels of E-selectin in CSF samples and total-tau/Aβ42 ratio in AD patients.
	- Järemo et al. [69]	- Blood from AD patients showed higher levels of P-selectin.

### 4.2. Vascular Damage

#### 4.2.1. Vascular Endothelial Cadherin (VE-Cadherin)

VE-cadherin is a CAM that is expressed in endothelial cells where it mediates the interaction between adjacent cells, making it a key protein to control vascular leakage [70]; therefore, elevated plasma levels of soluble VE-cadherin are indicative of vascular damage and dysfunction, as well as alterations in the BBB [71,72]. Interestingly, Aβ induces the release of the extracellular domain of VE-cadherin, and, so, elevated plasma levels of soluble VE-cadherin have been detected in AD patients, indicating vascular and BBB damage [72] (Figure 2). This blood biomarker could be used as an indicator of cognitive impairment as well, as its plasma levels were negatively correlated with the MMSE score and positively correlated with the CDR [72] (Table 2).

#### 4.2.2. Albumin

Albumin represents one of the most used biomarkers to determine the degree of BBB leakage by measuring the ratio CSF/serum-plasma albumin [73] (Figure 2). Given that albumin is a protein only synthesized in the liver, its presence in the CSF means BBB damage and eventually diffusion from the blood to both the CSF and the brain [73]. Several studies showed that AD patients have elevated ratios of both CSF/plasma [73,74] and CSF/serum [75,76,77] albumin compared to controls. Notably, a meta-analysis of data from 796 AD patients discovered that those with late-onset AD (disease onset over 65 years of age) had a significantly higher CSF/serum albumin ratio than controls, and this ratio was also positively correlated with the concentration of both Aβ_42_ and neurofilament light chain (NFL) (a biomarker of neuronal damage) [77]. Conversely, another meta-analysis from 854 AD patients found that albumin had a small effect size as a biomarker [76], which suggests that it is not useful for the diagnosis of the disease, but it is suitable for measuring BBB leakage (Table 2).

#### 4.2.3. Soluble Platelet-Derived Growth Factor Receptor-β (sPDGFRβ)

Another important marker of BBB damage is the sPDGFRβ [78]. High levels of this molecule, either in CSF or serum, reflect the release of the soluble fragment of the PDGFRβ from pericytes, a situation that occurs in the presence of ischemia or Aβ peptide [79] (Figure 2). Recently, Miner and colleagues have observed that the CSF concentration of sPDGFRβ is elevated in AD patients compared to controls, and it correlated with the levels of markers for AD progression, such as p-tau and T-tau in the CSF [30]. Furthermore, they also looked at the presence of albumin in the CSF and found that sPDGFRβ levels correlated with albumin ones in CSF samples [30] (Table 2); therefore, this may be indicating that levels of both markers in the CSF can be used as a rate of cerebrovascular damage and/or its progression in AD.

#### 4.2.4. Vasoactive Molecules

The atrial natriuretic peptide (ANP) is a cardiac hormone involved in the regulation of blood pressure and volume by promoting renal natriuresis and diuresis, as well as in vasodilation through the relaxation of vascular smooth muscle [80]. In addition, adrenomedullin (ADM) is a peptide that is involved in angiogenesis and cardiovascular homeostasis, and it also exhibits vasodilatory properties [81]. As previously mentioned, ET-1 is a paracrine hormone that acts as a vasoconstrictor primarily, but also as a vasodilator occasionally [82]. In this regard, ET-1 binds to the ET_B_ receptor on smooth muscle to stimulate vasoconstriction, while binding to the ET_A_ receptor on the endothelium releases vasodilatory substances, such as NO [82]. Therefore, all these three vasoactive molecules are considered biomarkers for several diseases associated with vascular dysfunction [80,81,82,83] (Figure 2). Interestingly, plasma samples from AD patients have shown that levels of pro-ADM and pro-ANP were increased compared to healthy subjects, in contrast to the levels of C-terminal ET-1 precursor fragment (CT-proET-1) which were decreased [83]. Controversially, elevated levels of ET-1 were also detected in AD brain samples [84]. Moreover, the pro-ANP/CT-proET-1 ratio was significantly elevated in patients with AD, displaying a diagnostic specificity and sensitivity of 80% and 72%, respectively [83]. This ratio may indicate a higher rate of vasodilatation versus vasoconstriction, highlighting a possible compensatory mechanism against vascular alterations seen in AD. In addition, the brain natriuretic peptide (BNP) is a hormone secreted by the cardiac ventricles. Among its functions, blood pressure regulation and cardiac remodeling are two of the most important [85]. Furthermore, elevated plasma levels of BNP are related to hypoxia and are an independent predictor of endothelial function [86,87]. Marksteiner and colleagues found higher plasma levels of N-terminal pro-BNP (BNP precursor) in both AD and MCI patients than in controls [88] (Table 2).

Homocysteine, a byproduct of methionine, is considered another important biomarker of endothelial dysfunction [89]. Elevated plasma levels of this molecule induce decreased levels of NO by several pathways, leading to endothelial dysfunction [90,91]. Recently, a meta-analysis has found both significantly increased plasma levels of homocysteine and decreased levels of folic acid in AD patients compared to controls [91]. Since folic acid and vitamin B12 are needed to re-methylate homocysteine and obtain methionine, low levels of either of them promote an increase in those of homocysteine and, thus, a higher risk of endothelial dysfunction [90,91]. Likewise, every increase of 5 µmol/L in plasma levels of homocysteine was associated with a 12% increased risk of AD [91]. Importantly, homocysteine levels can be lowered by dietary supplementation of folic acid and vitamin B [92], opening a new approach to the prevention of endothelial dysfunction and AD (Table 2).

Overall, studies focusing on vasoactive molecules brought inconclusive results. Whereas evidence in cardiac hormones may suggest that there is a trend to decrease vasoconstriction and increase vasodilatation in AD, studies on homocysteine seem to show the opposite; therefore, more studies are needed to fully elucidate the adaptive mechanism balancing the vascular dysfunction present in AD patients.

#### 4.2.5. Metalloproteinases (MMPs)

MMPs are endoproteinases that can degrade nearly all components of the extracellular matrix [93]. In addition, MMPs regulate proinflammatory processes and BBB leakage by inducing the cleavage of proinflammatory mediators, tight junction proteins, and basal lamina components [93] (Figure 2). Interestingly, certain MMPs, such as MMP-2, 3, and 9, exhibit a global Aβ-degrading activity, which is thought to reduce amyloid plaques [93]; however, microglia may enhance Aβ accumulation through an MMP-9-dependent mechanism [93]. Several alterations in the levels of MMPs have been detected in both CSF and plasma samples from AD patients [94,95,96,97,98,99]. Each MMP displayed different results in either CSF or plasma samples of AD patients: whereas the concentration of MMP-2 was significantly reduced [95], higher levels of MMP-3 were detected when compared to either MCI or controls [95,96]. Interestingly, the levels of MMP-3 correlated positively with CSF levels of T-tau and p-tau, and negatively with cognitive impairment [96]. Of note, cognitively normal patients with either AD-associated risk markers (e.g., T-tau, p-tau, and Aβ_42_ levels in the CSF) or the APOE4 allele showed elevated CSF levels of the MMP-3/Tissue inhibitor of metalloproteinase 1 (TIMP-1) ratio compared to counterparts without risk factors [97]. In contrast, the results regarding MMP-9 levels are controversial. On the one hand, Lorenzl and co-workers observed that plasma samples from AD patients showed higher levels of MMP-9 [94], but lower ones in the study developed by Horstman and colleagues [95]. This difference could be explained by the sample size as the former study analyzed more than twice the samples than the latter. On the other hand, CSF samples from AD patients did not show significant changes in MMP-9 levels in AD compared to control subjects [96,98]. Peptidylprolyl isomerase A, also known as cyclophilin A (CypA), facilitates protein folding and activates MPP-9 (Figure 2). A recent study has shown that plasma levels of CypA correlate with the MMSE score and global grey-matter volume (GMV) in AD patients positively [100]. In this regard, CypA may be indirectly involved in BBB disruption [99]. Besides MMP-3 and 9, increased levels of MMP-10 were detected and correlated with T-tau concentration in CSF samples from AD patients [98] (Table 2).

Finally, the action of metalloproteinases also promotes the release of the endothelial cell protein C receptor (EPCR) into the blood, which is physiologically present in vascular endothelial cells [101,102,103]. The binding of protein C to the receptor present in cells has anticoagulant functions, limits the inflammatory response, exerts a protective role at the vascular endothelial barrier, and reduces endothelial cell apoptosis in response to proinflammatory cytokines and ischemia [101,102] (Figure 2); however, when EPCR is soluble, it competes with cellular EPCR for protein C binding and, hence, the binding of protein C to sEPCR has an indirect pro-apoptotic, pro-coagulant, and pro-inflammatory function [101,102,103]. Interestingly, elevated serum levels of sEPCR were detected in AD patients, and they were positively correlated MMSE scores [104].

#### 4.2.6. Blood Coagulation System

Fibrinogen is a glycoprotein that is mainly involved in coagulation, as well as vasoreactivity and vascular permeability, inducing endothelial dysfunction at high concentrations [105] (Figure 2). In fact, plasma levels of fibrinogen are elevated in cardiovascular and cerebrovascular diseases [105], as well as in AD [106]; however, a later study found no significant differences in plasma levels of fibrinogen from AD samples compared to those in controls [107]. Despite this, correlations between fibrinogen levels and different AD biomarkers were found: (1) plasma concentration of fibrinogen was positively correlated with plasma concentration of Aβ_40_ and Aβ_42_ as well as CSF concentration of T-tau and p-tau-181, but negatively correlated with CSF concentration of Aβ_42_; (2) plasma concentration of fibrinogen was positively correlated with T-tau/Aβ_42_ and p-tau/Aβ_42_ ratios [107] (Table 2).

Plasminogen activator inhibitor-1 (PAI-1) is a regulator of the fibrinolytic system by inhibiting tissue-plasminogen activator (t-PA), eventually promoting clot formation [108]. PAI-1 is used as a biomarker of endothelial dysfunction and senescence [109]. Of interest, plasma levels of PAI-1 progressively increase in patients with either MCI or AD [110]. Thrombomodulin has been proposed as a BBB damage biomarker, since it is able to disrupt the vascular barrier in tissues other than CNS [111]. Interestingly, serum levels of the soluble thrombomodulin antigen were observed to be elevated in AD patients compared to controls [111] (Table 2).

**Table 2:** Main studies assessing biomarkers for vascular damage in AD.

Biomarkers	Publications	Results
VE-cadherin	- Lee et al. [72]	- AD patients had elevated plasma levels of soluble VE-cadherin, and those were negatively correlated with the MMSE score and positively correlated with the CDR.
Albumin	- Musaeus et al. [73], and Janelidze et al. [74]	- Elevated ratio of CSF/plasma albumin in AD patients.
	- Olson et al. [76]	- Small effects size of albumin as a biomarker for AD.
	- Skillbäck et al. [77]	- Higher ratio of CSF/serum albumin in AD patients than in controls, which was also positively correlated with the concentration of both Aβ42 and neurofilament light chain.
sPDGFRβ	- Miner et al. [30]	- Samples of CSF from AD patients showed increased levels of sPDGFRβ, that were correlated with the levels AD progression markers, such as p-tau and T-tau in the CSF.
Vasoactive molecules	- Buerger et al. [83]	- Increased levels of pro-ADM and pro-ANP but decreased levels of CT-proET-1 in plasma samples from AD patients. - The pro-ANP/CT-proET-1 ratio was also higher in AD patients and displayed a diagnostic specificity and sensitivity of 80% and 72%, respectively.
	- Marksteiner et al. [88]	- Both MCI and AD patients had higher plasma levels of N-terminal pro-BNP than controls.
	- Wang et al. [91]	- Elevated levels of homocysteine in plasma samples from AD patients. - Every increase of 5 µmol/L in plasma levels of homocysteine was associated with a 12% increased risk of AD.
MMPs	- Lorenzl et al. [94]	- High levels of MMP-9 in plasma samples from AD patients.
	- Horstmann et al. [95]	- Significant reduction in the plasma concentration of MMP-2 and MMP-9 in AD patients. - Higher plasma levels of MMP-3 than those from either MCI or control subjects.
	- Hanzel et al. [96]	- MMP3 levels correlate positively with CSF levels of T-tau and p-tau, but negatively with cognitive impairment. - No significant changes in the CSF concentration of MMP-9 from AD samples compared to controls.
	- Stomrud et al. [97]	- Cognitively normal patients with either AD-associated risk markers or the APOE4 allele showed elevated CSF levels of the MMP-3/Tissue inhibitor of metalloproteinase 1 (TIMP-1) ratio compared to counterparts without risk factors.
	- Bjerke et al. [98]	- CSF samples from AD patients displayed increased levels of MMP-10, which were correlated with T-tau concentration.
	- Zhu et al. [104]	- Increased serum levels of sEPCR were detected in AD patients, showing a positive correlation with MMSE scores.
Fibrinogen	- Fan et al. [107]	- Different correlations between plasma levels of fibrinogen and several AD biomarkers: - Plasma concentration of fibrinogen was positively correlated with plasma concentration of Aβ40 and Aβ42 as well as CSF concentration of T-tau and p-tau-181, but negatively correlated with CSF concentration of Aβ42. - Plasma concentration of fibrinogen was positively correlated with T-tau/Aβ42 and p-tau/Aβ42 ratios.
PAI-1	- Oh et al. [110]	- Progressive increase in plasma levels of PAI-1 in both MCI and AD patients

### 4.3. Growth Factors

#### 4.3.1. Vascular Endothelial Growth Factor (VEGF)

VEGF is a growth factor primarily involved in vasculogenesis and angiogenesis, and it is essential for proper vascular homeostasis [112,113]; moreover, VEGF is involved in adult hippocampal neurogenesis and hippocampal-dependent memory by increasing synaptic strength [114,115]. However, under ischemic conditions, VEGF also has harmful effects on the central nervous system by promoting BBB leakage in the brain [116] (Figure 2).

There is great controversy about the relevance of VEGF levels in both serum and CSF samples from AD patients. On the one hand, different studies from Mateo and colleagues and Li Huang and co-workers detected significantly lower serum levels of VEGF in AD patients compared to controls [117,118]. On the other hand, other studies reported an increase in serum levels of VEGF [119,120]. Indeed, elevated VEGF levels were associated with better memory and language performance in APOE4 carriers with severe–moderate AD [119]. Finally, other recent studies found no significant differences in serum levels of VEGF in AD patients compared to controls [121,122], although significantly higher levels have been detected in MCI patients [121,122] (Table 3).

Regarding CSF samples, either elevated [74,123,124], decreased [125] or unchanged [126] levels of VEGF have been detected. Accordingly, those three different outcomes have evidenced different correlations with cognitive impairment and altered in neuroimaging studies. For example, high CSF VEGF levels were associated with lower cognitive impairment, larger hippocampal volume, and less hippocampal atrophy [123]. This aligns with another study showing that elevated VEGF levels were associated with better executive function and episodic memory, and with a higher mean bilateral FDG-PET signal in the inferior parietal cortex and middle and inferior temporal gyrus [124]. Both works support previous reports suggesting that the relationship between elevated VEGF levels, improved cognitive function, and fewer anatomical and physiological alterations could be explained by the beneficial effect of VEGF on the hippocampus [114,115]; however, the combination of VEGF levels, the 3 classical CSF biomarkers of AD (Aβ_1-42_, T-tau, and p-tau), and another marker (heart-type fatty acid binding protein) displayed a sensitivity of 82.61% and specificity of 85.71% to distinguish between AD and controls [125]. Interestingly, patients with a CSF concentration of Aβ_42_ ≤ 192 pg/mL had a VEGF concentration in the CSF significantly lower compared to those patients with higher CSF Aβ_42_ levels [124] (Table 3).

Overall, more studies are mandatory to fully elucidate the relationship between VEGF and AD. Some explanations for the contradictory results may be the difference in sample size and the presence of depression in the patients (it has been previously reported that AD patients with depression have higher VEGF serum levels than those without [127]).

#### 4.3.2. Angiogenin

Angiogenin is a protein that induces endothelial proliferation, migration, invasion, and tube formation, thus participating in angiogenesis [128] (Figure 2). Beneficial roles have been suggested following hypoxia [129] and in neurodegenerative diseases [130]; however, the levels of angiogenin have yielded controversial results in the AD field. Kim and colleagues detected significantly reduced levels of angiogenin in the serum of AD patients compared to controls, and these reduced levels were associated with worse cognitive function [120]. Conversely, another analysis revealed elevated plasma levels of angiogenin in AD patients that were associated with a worse cognitive function [131] (Table 3). Of note, the patient database in the latter study was 10 times higher than the one in the former study. Nevertheless, further studies are needed to understand the role of this growth factor during AD pathogenesis.

#### 4.3.3. Angiopoietin-1 (Ang-1)

Ang-1 is a growth factor involved in angiogenesis, neurogenesis, and vascular protective functions, such as inhibition of endothelial apoptosis, suppression of inflammatory genes, and inhibition of vascular leakage [132,133] (Figure 2). Analysis of AD patients’ serum showed elevated levels of Ang-1 which were inversely correlated with MMSE scores [134]. Recently, it has been shown that Ang-1 accelerated the disease progression by promoting the formation of amyloid plaques and Aβ_42_, and increasing memory and learning impairment in the APP/PS1 transgenic mouse model [135]; however, it has been suggested that hypoxia might underlie such increases in Ang-1 levels in AD, since this pathological condition induces an increase in the expression of this growth factor [134] (Table 3).

**Table 3:** Studies assessing growth factors as biomarkers for AD.

Biomarkers	Publications	Results
VEGF	- Mateo et al. [117] and Huang et al. [118]	- Lower serum levels of VEGF in AD patients compared to controls.
	- Álvarez et al. [119] and Kim et al. [120]	- There is an increase in serum levels of VEGF in AD patients, which were associated with better memory and language performance in APOE4 carriers with severe–moderate AD.
	- Callahan et al. [121] and Schipke et al. [122]	- No significant differences in serum levels of VEGF in AD patients compared to controls. - Significant higher levels in MCI patients.
	- Guo et al. [125]	- The combination of VEGF levels, the three classical CSF biomarkers of AD (Aβ1-42, T-tau, and p-tau), and another marker (heart-type fatty acid binding protein) displayed sensitivity of 82.61% and a specificity of 85.71% to distinguish between AD and control subjects.
	- Hohman et al. [123]	- High levels of VEGF in the CSF were associated with lower cognitive impairment, higher hippocampal volume, and less hippocampal atrophy.
	- Tubi et al. [124]	- Elevated VEGF levels were associated with better executive function and episodic memory, and with a higher mean bilateral FDG-PET signal in the inferior parietal cortex and middle and inferior temporal gyrus.
Angiogenin	- Kim et al. [120]	- Reduced levels of angiogenin in the serum of AD patients compared to controls, which were associated with worse cognitive function.
	- Qin et al. [131]	- Elevated plasma levels of angiogenin in AD patients were associated with a worse cognitive function.
Ang-1	- Schreitmüller et al. [134]	- Levels of Ang-1 were increased in serum samples from AD patients, and they were inversely correlated with MMSE scores.

### 4.4. Blood Cells and Microparticles

Analysis of blood cells by flow cytometry can easily be used as a biomarker. Although this method of biomarker analysis is interesting and would be an easy technique to apply in the routine diagnosis of AD, there is no consistency between the results from studies currently available. In the AD field, circulating progenitor cells (CPCs), hematopoietic progenitor cells (HPCs), and endothelial progenitor cells (EPCs) are the main cells studied [1,2,136] (Figure 2). CPCs are a group of cells involved in tissue maintenance and repair, including HPCs and EPCs that are associated with vascular repair, their main marker being CD34^+^ [1,136]. Elevated levels of CPCs have been detected in AD patients, and this was correlated with cognitive decline [137,138]. However, other studies have found a reduction in CPCs in MCI and AD patients [139,140], that correlated with the CSF Aβ_42/40_ ratio and Aβ_1-42_ levels, as well as with hippocampal hyperperfusion, lower cortical thickness of the lower posterior cingulate gyrus, and memory alterations [139,140]. Similarly, studies addressing HPCs (CD34^+^/CD133^+^) levels show antagonistic results. A work from Stellos and colleagues found high levels of HPCs in AD patients that were inversely correlated with cognition [137]. In contrast, other studies showed lower levels of HPCs in samples from MCI and early AD patients compared to controls [139,141]. Regarding EPCs (CD34^+^/CD133^+^/KDR^+^), either lower [139,142] or unchanged [143,144] numbers of these cells have been detected in both AD and MCI patients, although the reduction in EPC numbers was correlated with increased cognitive impairment [142]. Remarkably, the properties of EPCs derived from patients in culture were altered when compared to those cells extracted from controls (e.g., reduction in migration, adhesion and chemotaxis, reduction of colony formation) [144,145,146]. The interaction between Aβ_1-42_ and EPCs has been suggested as an explanation for these modifications [146].

In recent years, microparticles analysis from plasma and blood samples by flow cytometry was able to detect membrane fragments derived from apoptotic cells or different cellular processes. Among microparticles being studied currently, endothelial microparticles (EMPs), derived from endothelial cells, appear as a promising option by indicating endothelial injury (Figure 2). Moreover, EMPs are elevated in different vascular pathologies, such as acute coronary syndromes or hypertension, among others [147]. In AD, plasma levels of EMPs are elevated compared to controls, and they correlate with cognitive impairment [148]; however, no significant differences in EMP levels between AD patients and controls have been recently reported [143].

## 5. Concluding Remarks

The main goal of this review was to compile the most remarkable and recent studies addressing different vascular-related molecules and cells as potential biomarkers or therapeutic targets in AD. Evidence from post-mortem AD brains show a feasible connection between vascular alterations and AD pathophysiology, which puts endothelial dysfunctions under the AD field spotlight to search for new therapeutic targets and/or disease-evolution biomarkers. Several biomarkers for vascular inflammation (e.g., OPN and CAMs) and vascular damage (e.g., albumin and MMPs) are being deeply studied in the AD field. Likewise, different growth factors (e.g., VEGF) and the analysis of cells and microparticles are rising in the AD forefront; however, many cellular mechanisms are not fully understood and, hence, more studies are still needed to elucidate the underlying pathways.

Overall, and despite controversy, endothelial damage associated with AD is undeniable at the structural level; therefore, this is an emerging field that demands more research to fully understand and decipher the mechanisms underlying the different connections between vessels, endothelial cells, and AD. In the future, AD-associated vascular changes have an enormous potential for revealing new therapeutic targets and/or biomarkers allowing the early diagnosis of AD.
